# Supplementary material for: Postpartum maternal bonding scale: Development and validation in a low- and middle- income country setting
Source: PLoS One. 2025 Apr 21;20(4):e0317936. doi: 10.1371/journal.pone.0317936 (PMC12011246; doi:10.1371/journal.pone.0317936)
Supplement: S1 Table — (PDF) [file pone.0317936.s001.pdf]

**S1 Table: Table of review of maternal bonding related instrument**

| S.no | Tool's name | Definition | Theoretical framework | Timeframe for assessment | Outcome of the scale | No. of items | Reliability | validity | Tool's strengths and limitations |
|---|---|---|---|---|---|---|---|---|---|
| 1 | Mother-to-Infant Bonding Scale (MIBS)[1] | feelings of the mother towards her infant | No explicit mention of a theoretical framework (items were based on their previous clinical work to determine bonding disorder in mothers) | Assessed mothers at 3 days and then at 12 weeks postpartum. Tool can be used from day one postpartum | Assesses failure to bond | 8 | Cronbach's Alpha was 0.71.<br>Stability of bonding and bonding scores at 3 days correlated significantly with those in the first few weeks and with those at week 12 | Negative affect correlated with poorer bonding and positive affect correlated with better bonding<br><br>Significant correlation between bonding and depression. | Strength:<br>-brief instrument<br><br>Limitations:<br>-Focus only on emotional aspect of bonding.<br>-No explicit mention of a theoretical framework. |
| 2 | Postpartum bonding questionnaire (PBQ)[2] | Bonding disorder has been defined as delay in or loss of maternal emotional response, pathological anger towards infant and rejection of infant.<br><br>Bonding disorder has been captured via 4 scales; impaired bonding, rejection and anger, anxiety and abuse. | No explicit mention of a theoretical framework (items were developed primarily based on the experiences of the mothers regarding their relationship with their babies) | Up to 12 weeks postpartum | Assesses bonding disorder | 25 | Correlation coefficients for the scale scores between the first and second administration of the instrument were 0.95, 0.95, 0.93 and 0.77 for the four scales. | PCA was done to identify 4 factors/scales. They identified sensitivity and specificity of the scales to identify mothers with low and high bonding disorders. Only scale 2 discriminates between mild and severe bonding disorder | Strength:<br>-Factor analysis was done to identify domains<br>-Cut off points were identified<br><br>Limitations:<br>-Focus on bonding disorder.<br>-No explicit mention of a theoretical framework. |
| 3 | Pre and postnatal bonding scale (PPBS)[3] | maternal emotions and feelings towards the fetus/infant | No explicit mention of a theoretical framework (Items generated based on past researches defining mothers positive emotions towards the child (fetus)) | Women completed the scale at 32 weeks' gestation. Same women completed the scale at eight and 12 months postpartum. | Assesses feelings of bonding | 5 same items were used in both prenatal and postnatal phases, with some modifications in the instructions | Cronbach alpha's at 32 weeks' gestation and at eight and 12 months postpartum were: 0.87, 0.80 and 0.79, respectively. Test-retest correlations of the PPBS at 32 weeks' gestation and at eight and 12 months postpartum were high: 0.42 and 0.41, and 0.67 between eight and 12 months | EFA was done at different time intervals; 32 weeks gestation, 8 and 12 months postpartum<br><br>CFA confirmed 5 items.<br><br>At 32 weeks' gestation, the PPBS correlated significantly with partner support (TPDS): 0.38. and depression (EDS): -0.24. Similar correlations with depression were | Strength: Brief scale.<br>EFA and CFA were conducted.<br><br>Limitations:<br>-Same items used for pre and postnatal phases<br>-Focus only on affective aspect of bonding.<br>-No mention of a theoretical framework |

| | | | | | | | | | |
|---|---|---|---|---|---|---|---|---|---|
| | | | | | | | postpartum, respectively. | found at eight (-0.23) and 12 months postpartum (-0.25). | |
| 4 | Parental Bonding Instrument (PBI)[4] | Not explicitly mentioned in the paper | Not explicitly mentioned. However authors mentioned that based on the literature review they identified two factors related to parental attitude and behaviour towards the child; care and psychological control over the child. | People were 20 to 61 years of age who responded retrospectively about their parents' behaviour during their first 16 years of their life | Assesses parental bonding in terms of care and over-protectiveness. | 25 | Test retest reliability of care scale was 0.761 and 0.628 for the 'overprotection' scale.<br>Split half reliability was 0.879 for the 'care' scale and 0.739 for the 'overprotection' scale. | Concurrent validity was determined via raters' scores of 'care ' and overprotection ' obtained at interview and those determined by the scales. There was significant association for the two care measures. | Strength: Concept of bonding was used but assessed retrospectively in adults<br><br>Limitations:<br>-tool used retrospectively. Adults respond about their parents' behavior during their first 16 years of their life.<br><br>-Not meant for prenatal or postpartum period<br><br>-No mention of definition<br>-No mention of theoretical framework |
| 5 | Maternal postanal attachment scale (MPAS)[5] | Emotional bond or tie of affection experienced by the parent towards the infant | They measured the construct of attachment by using theoretical model based on their previous framework to assess fetal attachment during pregnancy. They proposed four domains; (a)Pleasure in proximity; (b) Tolerance (c) Need gratification and protection (d) Knowledge acquisition | Authors conducted the assessment of mothers at 4 weeks, 4 months and 8 months postnatal<br><br>Tool can be used during first postnatal year | Assesses parent to child attachment | 19 items | Cronbach's alpha at three postnatal assessments 4 weeks, 4 months and 8 months postpartum ranges from 0.78 to 0.79 Test-retesr reliability via intraclass correlation coefficient was 0.70 | EFA was done and instead of 4, 3 factors were identified.<br><br>Maternal depression, anxiety and anger and social support were correlated with attachment. Child temperament was correlated with attachment | Strength:<br>Factors analysis was done.<br><br>Limitation:<br>-Used fetal attachment framework.<br>-Focus on attachment<br>-confusion of the term attachment vs bonding<br>-Definition not matched with the theoretical framework |
| 6 | mother-infant attachment scale[6] | Authors explained the concept as "attachment is described | No explicit mention of a theoretical framework (They developed items | mothers were interviewed within six months of the birth of the child | Assesses mother attachment | 15 | Split half reliability was done; the odd even items | Attachment of mother was less for babies separated | Strength: Brief scale |

| | | | | | | | | | |
|---|---|---|---|---|---|---|---|---|---|
| | | as an enduring relationship between parent and child within which they can interact positively and negatively, secure in the knowledge that their love for each other will remain intact and that each others well being is of prime importance.” | based on the opinion of pediatricians, psychologists and sociologists. They developed primarily two clusters (a)attachment and (b) expectation of mother from the child | | | | correlation was 0.83 after correcting for the length. The correlation of each half with the total score was 0.90 and 0.87 for odd and even items, respectively. | from them for more a week as compared to those who were separated less than a week (longer the separation lesser was the attachment)<br><br>Content validity was established based on the items' review by the experts. | Limitations:<br>-No explicit mention of theoretical framework.<br>-Focus on the concept of attachment |
| 7 | Mothers’ Object Relations Scales (MORS_SF)[7] | Not explicitly mentioned (tool focuses on mother's perception of child based on her internal working model) | It assess attachment. It was based on Psychodynamic approach focusing on internal working model that mother has about her infant. Using the items, they conducted PCA to have two factors invasion and warmth | Mothers were assessed when children were between2 and 6 months<br>- tool can be used upto 12 months postpartum | Assesses mother perception of infant's thoughts, behaviour towards her. It was measured via two scales; invasion and warmth | 14 | Test-retest reliability for Invasion scale was r = .77, and for the Warmth scale it was r = .70. Cronbach’s alpha values for both the Invasion and Warmth scales were .90, | Face validity: The items comprising the short-form were all derived from statements made spontaneously by mothers when describing their infants.<br><br>Factor analysis was done to have two factors warmth and invasion. Among the items in both factors, 14 higher loading items were identified to develop short 14 items scale. | Strength:<br>-Brief scale.<br>-Factor analysis was done.<br><br>Limitations: -Assesses attachment based on internal working model of psychodynamic approach.<br><br>-Definition not clearly mentioned |
| 8 | What Being the Parent of a New Baby Is Like (WPL-R)[8] | Three scales of this tools defined as Eval-uation (general satisfaction in being the parent of a new infant; Centrality (how much the infant, its care, or its physical health are on the parent’s mind; and Life Change (parent’s personal life and self-image, change in life and relationships with family | No explicit mention of a theoretical framework (focuses on experiences as a parent).<br>Items were developed based on open-ended questions with mothers. Based on this authors factor analysed the items and found three domains; evaluation, centrality and life change | Assessed mothers when child was 1 week, 1 month and 3 months old. | Assesses the parental image and parenting experience of parents of young infants via evaluation, centrality and life change | 25 | Cronbach’s Alpha at 1 week, 1 month, and 3 months were:.87, .90, and .87 for Evaluation; .87, .80 and .88 for centrality; and .77, .81 and .81 for Life Change.<br><br>Temporal stability was also explored | Factor analysis was done to obtain three factors; evaluation, centrality and life change.<br><br>Association between perceived competence and three domains of the scale was also | Strength: Factor analysis was done<br><br>Limiitation:<br>-Focus is only on experience of motherhood.<br>-Include items to assess relationship with other family members as well<br>-No explicit mention of |

| | | | | | | | | | |
|---|---|---|---|---|---|---|---|---|---|
| | | members, and overall stressfulness of life) | | | | | that were between in the range of moderate to high | obtained and were mostly significant. | theoretical framework |
| 9 | Being a mother scale (BAM-13)[9] | No explicit mention of the definition. Focus is on how does woman experience her motherhood | No explicit mention of a theoretical framework (Author developed items based on his clinical experience and observation and also reviewing general literature plus discussion with team of perinatal mental health experts. The author factor analysed the items and found 3 factors: child experience, adult experience, and emotional closeness) | Valid for women from the early postnatal weeks up to pre-school age (3–4 years postpartum) | Assesses satisfaction with the experience of being mother. It assesses it in three domains child experience, adult experience, and emotional closeness | 13 | Test-retest reliability was 0.52. Cronbach's Alpha was 0.798. Inter-item correlation matrix indicates that there was no item redundancy on the BaM-13, with no two items correlating greater than 0.55, and 73 of the 78 correlation coefficients being less than .5 | Correlation of this scale with depression was + 0.64<br>Cut off scores: Calculating the Receiver Operating Characteristics revealed that the optimal cut-off score on the BaM-13 against this criterion was 9 or more, giving a sensitivity of 72.5%, specificity of 74.4%, a positive predictive value of 34.9%, a negative predictive value of 93.5%, a misclassification rate of 25.9%, and a positive likelihood ratio of 2.83. The area under the curve was 0.81 (pb.001) | Strength:<br>-Brief scale<br>-Factor analysis was conducted.<br>Cut off scores were determined<br><br>Limitations:<br>-Focus only on the experience of motherhood.<br>- Includes items related to mothers experiences other than child such as loneliness, and lack of support.<br>-No explicit mention of the definition and a theoretical framework |

**Note:**

- This review was based on the parent tools (not based on the translated or adapted tools).
- Keywords related to bonding, postnatal, maternal, infant and tool were used to conduct search at pubmed. (Bond OR Bonding OR attachment) AND (maternal OR caregiver OR parent OR parental OR parents OR mother OR mother-infant OR mother and infant OR maternal-infant OR parent-infant) AND (postnatal OR postpartum OR post-partum) AND (tool OR scale OR inventory OR questionnaire OR instrument OR measure)
- Tool No. 1 to 4 assesses the construct of bonding; Tool No. 5 to 7 assesses the construct of attachment and tool no. 8 and 9 assess the experience of motherhood


1. Taylor A, Atkins R, Kumar R, Adams D, Glover V. A new Mother-to-Infant Bonding Scale: links with early maternal mood. Archives of women's mental health. 2005;8:45-51.
2. Brockington IF, Oates J, George S, Turner D, Vostanis P, Sullivan M, et al. A screening questionnaire for mother-infant bonding disorders. Archives of women's mental health. 2001;3:133-40.
3. Cuijlits I, Wetering AP VD, Potharst E, Truijens S, van Baar A, Vjm P. Development of a pre-and postnatal bonding scale (PPBS). Journal of Psychology & Psychotherapy. 2016;6(5).
4. Parker G, Tupling H, Brown LB. A parental bonding instrument. British journal of medical psychology. 1979.
5. Condon JT, Corkindale CJ. The assessment of parent-to-infant attachment: Development of a self-report questionnaire instrument. Journal of Reproductive and Infant Psychology. 1998;16(1):57-76.

6. Bhakoo O, Pershad D, Mahajan R, Gambhir S. Development of mother-infant attachment scale. Indian pediatrics. 1994;31:1477-.
7. Oates J, Gervai J. Mothers' perceptions of their infants. Journal of Prenatal & Perinatal Psychology & Health. 2019;33(4):282-300.
8. Pridham KF, Chang AS. What being the parent of a new baby is like: revision of an instrument. Res Nurs Health. 1989;12(5):323-9. Epub 1989/10/01. doi: 10.1002/nur.4770120508. PubMed PMID: 2798953.
9. Matthey S. Assessing the experience of motherhood: the Being a Mother Scale (BaM-13). Journal of affective disorders. 2011;128(1-2):142-52.
