# Supplementary material for: Postpartum maternal bonding scale: Development and validation in a low- and middle- income country setting
Source: PLoS One. 2025 Apr 21;20(4):e0317936. doi: 10.1371/journal.pone.0317936 (PMC12011246; doi:10.1371/journal.pone.0317936)
Supplement: S2 Table — (PDF) [file pone.0317936.s002.pdf]

S2 Table: List of Items in the 30-item Maternal Bonding Scale

#	Item
1	Do you affectionately touch your child?
2	Do you lovingly look at your child?
3	Do you try to spend more time with your child?
4	Do you look forward to be with your child?
5	Do you enjoy holding and picking up your child?
6	Do you feel love for your child?
7	Do you feel that you don't like your child?
8	Do you feel frustrated with your child?
9	Do you feel possessive towards your child?
10	Do you feel proud of your child?
11	Do you feel your child calms down when you pick him/her up?
12	Do you feel your child loves you when he/she looks at you?
13	Do you feel your child smiles when he/she looks at you?
14	Do you feel that your child exhausts you?
15	Do you feel your child needs you
16	Do you vocalize/talk with your child?
17	Do you respond (verbally) to your child's vocalizations?
18	Do you comfort your child when he/she cries?
19	Do you understand your child's signals?
20	Do you play with your child?
21	Do you feel that taking care of your child is your responsibility?
22	Do you look for information for your child's better development?
23	Do you feel that taking care of the child is a very difficult task?
24	Do you miss the life you had before this child?
25	Do you talk to others about your child?
26	Do you try to reorganize your life so that you could fulfill childcare responsibilities
27	Do you feel tense or anxious when you take care of your child?
28	Do you trust your own judgment when deciding what your child needs?
29	Do you feel trapped after becoming a mother?
30	Do you worry that you are not as good as other mothers?
