# Supplementary material for: Postpartum maternal bonding scale: Development and validation in a low- and middle- income country setting
Source: PLoS One. 2025 Apr 21;20(4):e0317936. doi: 10.1371/journal.pone.0317936 (PMC12011246; doi:10.1371/journal.pone.0317936)
Supplement: S3 Table — (PDF) [file pone.0317936.s003.pdf]

S3 Table: Comparison between study sample and general population:

Indicators	MICS[1] %	S-RMC %
**Women's age**		
Less than 20	8.8	9.6
20-34	75.6	69.3
35-49	15.7	21.1
**Women's education[2]**		
No education	77.4	82.4
Any formal education	22.6	17.6
**Mother tongue**	**Census 2023[3]**	
Sindhi - Thatta	92.9	88.1
**Household assets**		
Television	32.5	22.4
Refrigerator	14.2	11.2
Motorcycle, scooter, car other vehicle?	49.1	39.3
Open defecation (no facility, bush, field)	58.4	49.2
Improved source of drinking water	81.8	93.9
**Mental health**	**Systematic review[4]**	**S-RMC**
Pooled prevalence of postnatal depression	30	27.6

[1] Bureau of Statistics, Planning & Development Board, Government of Sindh. Sindh Multiple Indicator Cluster Survey Report 2018-19. Islamabad: Government of Pakistan & UNICEF; 2021 Jul.

[2] National Institute of Population Studies Pakistan, Macro International Inc. Pakistan Demographic and Health Survey 2017–18. Islamabad: Government of Pakistan; 2018

[3] Pakistan Bureau of Statistics. Census 2023.

[4] Atif M, Halaki M, Raynes-Greenow C, Chow CM. Perinatal depression in Pakistan: A systematic review and meta-analysis. Birth. 2021 Jun;48(2):149-163. doi: 10.1111/birt.12535
