# Supplementary material for: Postpartum maternal bonding scale: Development and validation in a low- and middle- income country setting
Source: PLoS One. 2025 Apr 21;20(4):e0317936. doi: 10.1371/journal.pone.0317936 (PMC12011246; doi:10.1371/journal.pone.0317936)
Supplement: S1 File — (PDF) [file pone.0317936.s004.pdf]

S4 File: Implementation guide and English and Urdu versions of Postpartum Maternal Bonding Scale (PMBS)

Implementation guide of Postpartum Maternal Bonding Scale (PMBS)

Definition of Postpartum Maternal Bonding: It is maternal or caregiver driven phenomenon that assesses mother's/caregiver's emotional, cognitive and behavioural capacity to connect with the child via emotional experiences with the child, understanding and responding to the child's needs and her sense of fulfilment and commitment to child care.

Purpose of the scale: The purpose of the scale is to measure the construct of maternal bonding with the child in the first year postpartum

Description of the scale: This scale has total 12 items that have been divided into three domains (4 items in each domain); Emotional: parent's emotional experiences and emotional display with the child (items 1-4), Cognitive: parent's perception and understanding of child's behaviour and needs and their capacity to respond to child's needs (items 5-8), and Behaviour: parent's sense of fulfilment and commitment to child care (items 9-12).

Respondent for the scale: Mothers in their first year of postpartum (who has an alive index child) would be the respondent for this scale. In case of unavailability of the mother, any primary caregiver can be the respondent. However we have not yet tested this instrument on caregivers other than mothers.

Format of assessment: Trained field staff can interview a mother using this scale. However, if needed, it may also be used as a self-report instrument.

Administration and scoring

1. Start the interview of the mother by giving her the following instructions: "I would like to ask a few questions about your feelings, thoughts and behaviour towards your child. Please respond with one of the following options: Never or one-off (0) Rarely (1) Some of the time (2) Most or all the time (3)
2. Ask each question one by one and record her responses.
3. To identify the score of bonding, tally the numbers of checked responses for all the items. For each domain: Emotional (questions 1 to 4), Cognitive (questions 5 to 8), and Behavioural (questions 9 to 12).
4. Add the numbers together to calculate the total postpartum maternal bonding score. Similarly, for each domain, add the numbers together to calculate the domain-specific score.
5. Please note items 9 to 12 will be reverse scored.

Interpretation

An increasing score in overall bonding and its domains indicates better maternal postpartum bonding with the child. However it is important to highlight that attaining zero score in any domain warrants particular concern, as it signifies a complete absence of that aspect of the bonding. Therefore, attention should be given to such cases, as well as to those with low scores– both overall and domain-specific.

Note: Both English and Urdu versions of Postpartum Maternal Bonding Scale (PMBS) have been included in this article to be used for research purposes and service provision only. Those who wish to use the scale are kindly requested to cite this paper. For adaptation and translations into other languages, please seek permission from the corresponding author.

Suggested citation: Khan B, Soremekun S, Hameed W, Avan BI. Postpartum Maternal Bonding Scale: development and validation in a low- and middle-income country setting. PLOS ONE. 2025

12-Item Postpartum Maternal Bonding Scale (PMBS) in English with Domains and Response options

Postpartum Maternal Bonding Scale			
**Instructions:** I would like to ask a few questions about your feelings, thoughts and behaviour towards your child. Please respond with one of the following options: Never or one-off, rarely, some of the time, or most or all the time			
**Q.no**	**Question**	**Response**	**Score**
**I. Emotional:** parent's emotional experiences and emotional display with the child			
1	Do you affectionately touch your child?	0 Never or one-off<br>1 Rarely<br>2 Some of the time<br>3 Most or all the time	
2	Do you enjoy holding and picking up your child?	0 Never or one-off<br>1 Rarely<br>2 Some of the time<br>3 Most or all the time	
3	Do you feel love for your child?	0 Never or one-off<br>1 Rarely<br>2 Some of the time<br>3 Most or all the time	
4	Do you feel possessive towards your child?	0 Never or one-off<br>1 Rarely<br>2 Some of the time<br>3 Most or all the time	
**II. Cognitive:** parent's perception and understanding of child's behaviour and needs and responding to child's needs			
5	Do you feel your child smiles when he/she looks at you	0 Never or one-off<br>1 Rarely<br>2 Some of the time<br>3 Most or all the time	
6	Do you vocalize/talk with your child?	0 Never or one-off<br>1 Rarely<br>2 Some of the time<br>3 Most or all the time	
7	Do you understand your child's signals?	0 Never or one-off<br>1 Rarely<br>2 Some of the time<br>3 Most or all the time	
8	Do you play with your child?	0 Never or one-off<br>1 Rarely<br>2 Some of the time<br>3 Most or all the time	
**III. Behaviour:** parent's sense of fulfilment and commitment to child care			
9	Do you feel that taking care of your child is a very difficult task	3 Never or one-off<br>2 Rarely<br>1 Some of the time<br>0 Most or all the time	
10	Do you miss the life you had before this child?	3 Never or one-off<br>2 Rarely<br>1 Some of the time<br>0 Most or all the time	
11	Do you feel trapped after becoming a mother?	3 Never or one-off<br>2 Rarely<br>1 Some of the time<br>0 Most or all the time	
12	Do you worry that you are not as good as other mothers?	3 Never or one-off<br>2 Rarely<br>1 Some of the time<br>0 Most or all the time	
	Note: The coding of items 9, 10, 11 and 12 has been intentionally reversed.		

**Domains**	**Scores**
I. Emotional (Item 1-4)	____ / 12
II. Cognitive (Item 5-8)	____ / 12
III. Behaviour (Item 9-12)	____ / 12
**Overall Bonding (Item 1-12)**	**____ / 36**

12-item Postpartum Maternal Bonding Scale (PMBS) in Urdu

Postpartum Maternal Bonding Scale			
**ہدایات:** میں آپ سے آپ کے بچے /بچی کے لیے آپ کے خیالات، جذبات اور رویوں کے بارے میں کچھ سوالات پوچھنا چاہوں گی۔ برائے مہربانی آپ ان چار جوابات میں سے کوئی ایک جواب دیں۔<br>کبھی نہیں یا ایک عاد بار، شاز و نادر (کم کم)، کبھی کبھی، اکثر یا ہمیشہ			
**سوال نمبر**	**سوال**	**جوابات**	**سکور**
**I. Emotional**: parent’s emotional experiences and emotional display with the child			
1	کیا آپ اپنے بچے/بچی کو شفقت سے چھوتی ہیں؟	0-کبھی نہیں یا ایک آدھ بار<br>1-شاز و نادر (کم کم)<br>2-کبھی کبھی<br>3-اکثر یا ہمیشہ	
2	کیا آپ کو اپنے بچے/بچی کو تھامنا/اُٹھانا اچھا لگتا ہے؟	0-کبھی نہیں یا ایک آدھ بار<br>1-شاز و نادر (کم کم)<br>2-کبھی کبھی<br>3-اکثر یا ہمیشہ	
3	کیا آپ اپنے بچے/بچی کے لئے پیار محسوس کرتی ہیں؟	0-کبھی نہیں یا ایک آدھ بار<br>1-شاز و نادر (کم کم)<br>2-کبھی کبھی<br>3-اکثر یا ہمیشہ	
4	کیا آپ محسوس کرتی ہیں کہ یہ بچہ/بچی صرف آپ کا /کی ہے اور کسی کا /کی نہیں؟	0-کبھی نہیں یا ایک آدھ بار<br>1-شاز و نادر (کم کم)<br>2-کبھی کبھی<br>3-اکثر یا ہمیشہ	
**II. Cognitive**: parent’s perception and understanding of child’s behaviour and needs and responding to child’s needs			
5	کیا آپ کو محسوس ہوتا ہے کہ آپ کا بچہ /بچی آپ کی طرف دیکھ کر مسکراتا / مسکراتی ہے؟	0-کبھی نہیں یا ایک آدھ بار<br>1-شاز و نادر (کم کم)<br>2-کبھی کبھی<br>3-اکثر یا ہمیشہ	
6	کیا آپ بچے /بچی سے باتیں کرتی ہیں/آوازیں نکالتی ہیں؟	0-کبھی نہیں یا ایک آدھ بار<br>1-شاز و نادر (کم کم)<br>2-کبھی کبھی<br>3-اکثر یا ہمیشہ	
7	کیا آپ اپنے بچے/بچی کے اشاروں کو سمجھتی ہیں؟	0-کبھی نہیں یا ایک آدھ بار<br>1-شاز و نادر (کم کم)<br>2-کبھی کبھی<br>3-اکثر یا ہمیشہ	
8	کیا آپ اپنے بچے/بچی کے ساتھ کھیلتی ہیں؟	0-کبھی نہیں یا ایک آدھ بار<br>1-شاز و نادر (کم کم)<br>2-کبھی کبھی<br>3-اکثر یا ہمیشہ	
**III. Behaviour**: parent’s sense of fulfilment and commitment to child care			
9	کیا آپ کو لگتا ہے کہ اپنے بچے/بچی کا خیال رکھنا ایک بہت مشکل کام ہے؟	3-کبھی نہیں یا ایک آدھ بار<br>2-شاز و نادر (کم کم)<br>1-کبھی کبھی<br>0-اکثر یا ہمیشہ	
10	کیا آپ کو اس بچے/بچی سے پہلے والی زندگی یاد آتی ہے؟	3-کبھی نہیں یا ایک آدھ بار<br>2-شاز و نادر (کم کم)<br>1-کبھی کبھی<br>0-اکثر یا ہمیشہ	
11	کیا آپ کو محسوس ہوتا ہے کہ آپ ماں بن کے پھنس گئی ہیں؟	3-کبھی نہیں یا ایک آدھ بار<br>2-شاز و نادر (کم کم)<br>1-کبھی کبھی<br>0-اکثر یا ہمیشہ	
12	کیا آپ پریشان ہوتی ہیں کہ آپ اور مائوں کی طرح اچھی ماں نہیں ہیں؟	3-کبھی نہیں یا ایک آدھ بار<br>2-شاز و نادر (کم کم)<br>1-کبھی کبھی<br>0-اکثر یا ہمیشہ	
	نوٹ: سوال نمبر ۹، ۱۰، ۱۱، ۱۲ دانستہ طور پر ‘reverse coding’ پر تبدیل کیے گئے ہیں۔		

Domains	Scores
I. Emotional (Item 1-4)	____ / 12
II. Cognitive (Item 5-8)	____ / 12
III. Behavioural (Item 9-12)	____ / 12
**Overall Bonding (Item 1-12)**	**____ / 36**
