# Supplementary material for: Postpartum maternal bonding scale: Development and validation in a low- and middle- income country setting
Source: PLoS One. 2025 Apr 21;20(4):e0317936. doi: 10.1371/journal.pone.0317936 (PMC12011246; doi:10.1371/journal.pone.0317936)
Supplement: Attachment — Submitted filename: Response to reviewers comments_Final 22Oct.docx [file pone.0317936.s006.docx]

**Point by point response to reviewers’ comments**

We are thankful to the reviewers for their very helpful and insightful comments. Following are point by point response to the comments of reviewer 1 and 2.

| **Reviewer 1** | | | |
| --- | --- | --- | --- |
| **S.No.** | **Reviewer’s Comment** | **Authors’ Response** | **Suggested changes incorporated**  **(Page and line no.). Please see revised manuscript with track changes** |
| 1a | Dear Editor thank you for your invitation to review manuscript entitled" Postpartum Maternal Bonding Scale: development and validation in a low- and middle- income country setting"  Overall, the introduction provides a solid foundation for the research.  It clearly outlines the significance of early maternal bonding, highlights the research gap in LMIC settings, and states the study objectives. However, there are a few areas where it could be strengthened:  Cultural Sensitivity:  -While the introduction mentions ensuring cultural relevance, it might be beneficial to elaborate on specific strategies used to adapt the scale to different cultural contexts.  - | Thank you so much for your encouraging comments  Cultural sensitivity is one of the recommendations for the measurement of latent constructs. We have already included detailed process in the procedure section. But as you have highlighted, we have mentioned key strategies pertaining to this process in this paragraph as well. | Please see ‘introduction’, under the heading of ‘cultural relevance’ , page 11, line: 226-227. |
| 1b. | Consider consulting with experts from the target LMICs to ensure the scale is culturally appropriate and sensitive. | Thanks for this comment. Consulting with experts from the LMIC to ensure cultural appropriateness was one of the key strategies of the model we used in our study. We have already added details of this strategy in the procedure and results sections.  As you have highlighted, we have mentioned it as one of the strategies in introduction as well | Please see ‘introduction’, under the heading of ‘cultural relevance’ , page 11, line: 228-229 |
| 2 | Strengthen the Thesis Statement:  -Consider refining the thesis statement to more directly state the purpose of the study. For example, you could state: "This study aims to develop and validate a postpartum maternal bonding scale specifically designed for LMIC settings, addressing the existing gaps in the literature." | Thanks for this point. We have revised the thesis statement. | Please see ‘introduction’, page 12, line: 242-244 |
| 3a | 3. Expand on the Literature Review:  While the literature review provides a good overview of existing instruments, it could be further enhanced by:  -Discussing the theoretical frameworks underpinning these instruments. | Thank you for highlighting this point. We would like to mention here that only two studies refer the use of theoretical/conceptual framework in the development of their instrument. We have clarified it in the write up and have included the details in the introduction section.  We have also included the relevant references;  18. Condon JT, Corkindale CJ. The assessment of parent-to-infant attachment: Development of a self-report questionnaire instrument. Journal of Reproductive and Infant Psychology. 1998;16(1):57-76.  22. Oates J, Gervai J. Mothers' perceptions of their infants. Journal of Prenatal & Perinatal Psychology & Health. 2019;33(4):282-300.  25. Goulet C, Bell L, St-Cyr D, Paul D, Lang A. A concept analysis of parent-infant attachment. J Adv Nurs. 1998;28(5):1071-81. Epub 1998/12/05. doi: 10.1046/j.1365-2648.1998.00815.x. PubMed PMID: 9840879. | Please see introduction, page 5, line 106-117 |
| 3b | -Comparing and contrasting the strengths and limitations of different scales. | Thank you for this. Review of existing instruments was an important factor in identifying the gap in literature and measurement pertaining to the concept of bonding and finally deciding to develop an instrument. We have already included this comparison in the introduction section and have also added some more content relevant to it.  In addition to this, a table of tool review (S1 Appendix-Table 1) has already been included in the supplementary file. We have also added one more column indicating strength and limitations of all the tools. | Please see introduction, page 5-6, line: 99-139  Please see also see S1 Appendix-Table 1 in the Revised Supplementary file |
| 3c | -Analyzing why these existing scales might not be fully applicable in LMIC settings. | Thank you for your comment. We have already included details about the aspects of the existing scales due which they might not be fully applicable to the LMIC settings. But as you have suggested we have further highlighted it in the write up. | Please see introduction, page 6, line: 127-133 |
| 4a | Clarify the Conceptual Framework:  -Explicitly define the concept of postpartum maternal bonding, including its key components and dimensions. | Thank you so much for your comment. We have explicitly added the definition of the maternal postpartum bonding and clearly explains the framework’s domains. | Please see introduction, page 9-10, line: 177-190 |
| 4b | -Discuss how this definition aligns with existing theoretical frameworks (e.g., attachment theory). | Thanks for this comment. We have mentioned it in detail in the manuscript. We have further explicitly clarified the overlapping and distinct elements of bonding and attachment.  We have also included additional references in the manuscript as listed below;  29. Bowlby J. The nature of the child's tie to his mother. Int J Psychoanal. 1958;39(5):350-73. Epub 1958/09/01. PubMed PMID: 13610508.  30. Ainsworth MDS, Blehar MC, Waters E, Wall SN. Patterns of attachment: A psychological study of the strange situation. Hillsdale, NJ: Erlbaum; 1978.  31. Klaus MH, Kennell JH. Mothers separated from their newborn infants. Pediatr Clin North Am. 1970;17(4):1015-37. Epub 1970/11/01. doi: 10.1016/s0031-3955(16)32493-2. PubMed PMID: 4922925.  32. Klaus MH, Jerauld R, Kreger NC, McAlpine W, Steffa M, Kennel JH. Maternal attachment. Importance of the first post-partum days. N Engl J Med. 1972;286(9):460-3. Epub 1972/03/02. doi: 10.1056/nejm197203022860904. PubMed PMID: 5009748. | Please see introduction, page 6-9, line: 135-172  Please also see ‘References’ reference no. 29,30,31,32 |
| 5a | Strengthen the Discussion of LMIC Context:  -Elaborate on the unique challenges and opportunities for maternal bonding in LMIC settings. | Thanks for this point. As you have suggested, we have added the relevant content in the manuscript.  We have also included additional references in the manuscript as listed below;  36. Roddy Mitchell A, Gordon H, Lindquist A, Walker SP, Homer CSE, Middleton A, et al. Prevalence of Perinatal Depression in Low- and Middle-Income Countries: A Systematic Review and Meta-analysis. JAMA Psychiatry. 2023;80(5):425-31. Epub 2023/03/09. doi: 10.1001/jamapsychiatry.2023.0069. PubMed PMID: 36884232; PubMed Central PMCID: PMCPMC9996459.  37. O'Dea GA, Youssef GJ, Hagg LJ, Francis LM, Spry EA, Rossen L, et al. Associations between maternal psychological distress and mother-infant bonding: a systematic review and meta-analysis. Arch Womens Ment Health. 2023;26(4):441-52. Epub 2023/06/15. doi: 10.1007/s00737-023-01332-1. PubMed PMID: 37316760; PubMed Central PMCID: PMCPMC10333415.  38. Delisle HF. Poverty: the double burden of malnutrition in mothers and the intergenerational impact. Ann N Y Acad Sci. 2008;1136:172-84. Epub 2008/06/27. doi: 10.1196/annals.1425.026. PubMed PMID: 18579881.  39. Britto PR, Lye SJ, Proulx K, Yousafzai AK, Matthews SG, Vaivada T, et al. Nurturing care: promoting early childhood development. Lancet. 2017;389(10064):91-102. Epub 2016/10/09. doi: 10.1016/s0140-6736(16)31390-3. PubMed PMID: 27717615.  40. Antelman G, Ferla J, Gill MM, Hoffman HJ, Komba T, Abubakar A, et al. Effectiveness of an integrated multilevel early child development intervention on caregiver knowledge and behavior: a quasi-experimental evaluation of the Malezi program in Tanzania. BMC Public Health. 2023;23(1):19. Epub 2023/01/05. doi: 10.1186/s12889-022-14956-2. PubMed PMID: 36600280; PubMed Central PMCID: PMCPMC9811787. | Please see introduction, page 10, line: 194-201  Please see ‘References’, reference no. 36, 37, 38, 39, 40 |
| 5b | -Discuss how cultural, social, and economic factors might influence the development and validation of the scale. | Many thanks. We have added the relevant details in the manuscript. | Please see ‘Introduction’, under the heading of ‘Suitable for use in LMIC’ page 12, line: 234-237 |
| 6a | Method  1. Sample Size and Representation:  • Discuss the representativeness of the sample in terms of age, education level, socioeconomic status, and other relevant demographic factors. | Thank you for the comment. We have added the relevant details in the manuscript. We have also added a new table ‘Table 2’ to illustrate socio-demographic details of the participants and also included a supplementary file. | Please see ‘Results’ section;  Please see new ‘Table 2’ with characteristics of the sample on page 26, and wordings to describe the sample on page 21, lines 454-459  A supplementary file )S3 Appendix, Table 3) has also been added. |
| 6b | • Consider providing more information on the recruitment process and inclusion/exclusion criteria. | Many thanks for your comment. We have added the relevant details in the manuscript. | Please see “Method” section, page 13, line: 257-276 |
| 7a | Cultural Adaptation Process:  • Elaborate on the specific strategies used to adapt the scale to the LMIC context, such as back-translation, cognitive interviews, and expert review. | Thanks for your comment. We primarily developed the maternal postpartum bonding scale. It was initially developed in English language and then it was translated into Urdu language and also in Sindhi language. We followed a thorough process. We have included the details related to translation, back-translation, cognitive interviews, expert review and pilot. | Please see “Result’ section, page 19-21, line: 400-450 |
| 7b | • Discuss any challenges encountered during the cultural adaptation process. | Thank you for this point. We have added the details in the result section. | Please see “Result” section, page 19, line: 407-415 |
| 8a | Psychometric Properties:  • Provide more details on the statistical techniques used for construct validity, reliability, and internal consistency analysis. | Thank you for highlighting this. We have added the details in the “Method” section of the manuscript.  Also, we have explained and interpreted the methods used to assess validity, reliability and internal consistency in the Results and Discussion sections. As all are standard methodologies, we have only provided references where appropriate. | Please see “Methods” section on page 18, under the heading of “Statistical analysis, lines: 379-384  Please see “Results” section pages 21-25, lines 463-548  Please see “Discussion” section page 36, Lines 663-668 |
| 8b | • Report the Cronbach's alpha coefficient and factor analysis results. | Thanks for your comments. Cronbach’s alpha and Omega coefficient and factor analysis results and their interpretations have already been included in the manuscript. Two additional references have also been added.  49. Tavakol M, Dennick R. Making sense of Cronbach's alpha. Int J Med Educ. 2011;2:53-5. Epub 2011/06/27. doi: 10.5116/ijme.4dfb.8dfd. PubMed PMID: 28029643; PubMed Central PMCID: PMCPMC4205511.  50. McNeish D. Thanks coefficient alpha, we'll take it from here. Psychol Methods. 2018;23(3):412-33. Epub 2017/05/31. doi: 10.1037/met0000144. PubMed PMID: 28557467. | Please see following in the “Results” section;  For Alpha and Omega coefficients:  -Please see page 24, Lines 527-529.  -References: Please see reference no. 49 and 50  -Please see Table 4b on page 30  For Factor Analysis:  -Please see page 21-24, Line 463-521  -Also please see Fig 3 |
| 9a | Results:  Factor Analysis and Model Fit:  -Provide more details on the criteria used to determine the number of factors to extract in the exploratory factor analysis (EFA). | Thank you for your comment. We have used best practice methods to determine number of factors to extract, this is already explained in the manuscript.  An additional reference has been added.  48. Williams B, Onsman A, Brown T. Exploratory Factor Analysis: A Five-Step Guide for Novices. Australasian Journal of Paramedicine. 2010;8:1-13. doi: 10.33151/ajp.8.3.93. | Please see “Results” section, pages 21-22, lines: 463-469  Please also see “References” reference no. 48 |
| 9b | -Discuss the implications of the bifactor model fit in terms of the scale's underlying structure and interpretation. | Thank you. We have discussed the meaning and interpretation of the bifactor model structure model in both the results and discussion sections. | Please see “Results” section, Page 23, Line 511-513  Discussion: page 36, Line 663-666 |
| 9c | -Report the full details of the confirmatory factor analysis (CFA) models, including the specific goodness-of-fit indices and their cut-offs. | Thank you for your comment. We have included an extensive description of the confirmatory factor analysis models and their goodness of fit test values and cutoffs in both the results and discussion sections. | Please see “Results” section;  -Page 22-24, Line 492-521  -Table 3 pages 27-29  -Figure 3  Please see “Discussion” section; -Page 36, Lines 666-668 |
| 10a | Cultural Adaptation and Translation:  Elaborate on the specific strategies used to ensure the equivalence of the scale across languages (e.g., back-translation, expert review). | Thank you for your comment. We have included the relevant details. | Please see “Result’ section, page 19-21, line: 400-450 |
| 10b | Discuss any challenges encountered during the cultural adaptation process and how they were addressed. | Thank you for this point. We had a particular challenge when we had to translate/adapt the instrument into two local languages. Therefore we handled it by engaging two independent linguists who had command over all required languages. We have added these details in the result section. | Please see “Result” section, page 19, line: 407-415 |
| 11a | External Validity:  Consider discussing the potential for external validity by;  -comparing the scale's findings to those from other studies or existing instruments. | Thank you. We have dedicated a section in the discussion for external validity comparisons with similar instruments, and have included an additional reference.  17. Taylor A, Atkins R, Kumar R, Adams D, Glover V. A new Mother-to-Infant Bonding Scale: links with early maternal mood. Archives of women’s mental health. 2005;8:45-51. | Please see “Discussion” section, page 37-38, Line: 693- 706  Please see “References”; Reference no. 17 |
| 11b | -Explore the generalizability of the scale to different populations and settings. | Thank you for this point. We have added the relevant content in the manuscript | Please see ‘Discussion’ section, page 40, line: 749-751 |
| **Reviewer 2** | | | |
| **S.No.** | **Reviewer’s Comment** | **Authors’ Response** | **Suggested changes incorporated**  **(Page and line no.)Please see manuscript with track changes** |
|  | Thank you for the opportunity to review the manuscript “Postpartum Maternal Bonding Scale: development and validation in a low- and middle-income country setting”. I read it with great interest, and I applaud the huge work the authors have done in developing and validating an instrument. Especially in a setting that hasn’t been studied much. However, I also have some comments for the authors: | Thank you for your encouraging words and valuable feedback. |  |
| 1 | Abstract  1. Ll. 55 “Regression analyses showed associations…”: The authors should state the direction of the associations. | Thank you for highlighting it. We have now added the direction of the associations as well as the model output in the Abstract. | Please see “Abstract”, Page 3 Line: 57-61 |
| 2 | 2. L. 61: I would be cautious not to overstate the instrument’s potential cross-cultural use, as the authors have only tested it in one region. | Thank you for your comment. We have specifically highlighted in discussion that instrument can be used after cultural adaptation. We have now mentioned it in the abstract as well. | Please see ‘Abstract’, page 3, line: 64-66 |
| 3 | Introduction  3. Ll. 118-119: I do not agree with the statement that attachment appears at 11-24 months. Rather, it is when it can be reliably assessed with the Strange Situation | Thank you for this comment. We have revised the statement in the introduction.  We have also made the correction in Table 1. | Please see ‘introduction’ section, page 7, line: 155-156  Please see Table 1, page 8 |
| 4 | 4. Table 1: I am unclear on what the authors mean with a lot of these differences between attachment and bonding, like caregiver consequences and child consequences. I do not see why those examples are particular to bonding and attachment. References are sorely needed. | Thank you for highlighting this. We have explained the differences between the constructs of bonding and attachment in the write up.  We have also included relevant references in the manuscript as listed below;  26. Le Bas G, Youssef G, Macdonald JA, Teague S, Mattick R, Honan I, et al. The Role of Antenatal and Postnatal Maternal Bonding in Infant Development. J Am Acad Child Adolesc Psychiatry. 2022;61(6):820-9 e1. Epub 2021/09/24. doi: 10.1016/j.jaac.2021.08.024. PubMed PMID: 34555489.  27. Bicking Kinsey C, Hupcey JE. State of the science of maternal-infant bonding: a principle-based concept analysis. Midwifery. 2013;29(12):1314-20. Epub 2013/03/05. doi: 10.1016/j.midw.2012.12.019. PubMed PMID: 23452661; PubMed Central PMCID: PMCPMC3838467.  28. Ettenberger M, Bieleninik Ł, Epstein S, Elefant C. Defining Attachment and Bonding: Overlaps, Differences and Implications for Music Therapy Clinical Practice and Research in the Neonatal Intensive Care Unit (NICU). Int J Environ Res Public Health. 2021;18(4). Epub 2021/02/14. doi: 10.3390/ijerph18041733. PubMed PMID: 33579015; PubMed Central PMCID: PMCPMC7916808.  30. Ainsworth MDS, Blehar MC, Waters E, Wall SN. Patterns of attachment: A psychological study of the strange situation. Hillsdale, NJ: Erlbaum; 1978.  33. Nakić Radoš S, Hairston I, Handelzalts JE. The concept analysis of parent-infant bonding during pregnancy and infancy: a systematic review and meta-synthesis. J Reprod Infant Psychol. 2024;42(2):142-65. Epub 2023/01/03. doi: 10.1080/02646838.2022.2162487. PubMed PMID: 36588501.  34. Berk LE. Exploring lifespan development. 4th ed. Boston: Pearson; 2018. | Please see ‘introduction’ section, page 7, line: 150-166  Please see ‘References” Reference no. 26,27,28,30,33,34 |
| 5 | 5.I am not sure I understand the difference between the emotional and behavioral domains in the authors’ figure. As an example of behavioral, the authors mention the mother’s sense of fulfilment, however, isn’t that also an emotion and could be in the emotional domain? The authors mention that this is based on a thorough review of the literature, but it would be helpful if the authors could review this literature more thoroughly in the manuscript as well. | Thank you for this comment. We have further elaborated the framework and domains in the revised manuscript.  To further clarify it, emotional domain focuses on the kind of emotions mother feels and displays towards her child whereas behavioural domains focuses on mother’s engagement, sense of satisfaction/fulfillment and commitment when she is involved in child care tasks. | Please see ‘introduction’, page 9-10, line: 177-190 |
| 6 | Results  6. Ll. 317: Could the authors give some examples of the items that were removed to create transparency in the process? | Thank you for highlighting this. We have included the some examples of items that were removed after cognitive interview and expert review. | Please see ‘Results’, page 20, line: 433-442 |
| 7 | 7. Ll. 386: What thresholds did the authors use to evaluate the goodness of fit estimates? I see some are mentioned in Table 2, but references on them would be good. | Thank you - we have included thresholds and references for all model goodness of fit tests in the Discussion section. | Please see “Discussion” section, page 36, line: 666-668 |
| 8 | 8. There are two Table 3s: One about correlations and reliability and one about descriptive statistics. | Thank you so much for highlighting it. We have made the corrections in the manuscript. We have also updated the numbers of all the tables. | Please see;  -Table 4a for correlation on page 30  -Table 4b for reliability on page 30  -Table 4c for descriptive statistics on page 31 |
| 9 | 9. Regarding the second Table 3 on the descriptive statistics and Figure 4 on the density distribution, I would be interested in knowing the distribution of scores on all the individual items. Do the authors see low scores on any of the items? Because if not, it may be an indication of the sample not being representative in giving low scores, challenging the validation of the instrument. | We reviewed the distribution of scores for all items and found responses in every category across all items. There were no items where any response category had a count of zero. | No changes have been made to the manuscript |
| 10 | 10. I would urge the authors to consider using item response theory to also choose which items to retain or not rather than just relying on expert/participant evaluations and loadings in the exploratory factor analysis. The strength of using item response theory is that it can be used to evaluate at which levels of the construct that the item discriminates. That way the authors can be sure that they have items that assess the construct at all levels of the trait. | Thanks for your comment. We used expert evaluations to ensure that items are conceptually, content wise and culturally relevant. We have elected to use EFA and CFA which are recommended and widely applied statistical techniques for instrument development. Item response theory (IRT) is a valuable statistical technique but it arguably lacks the conceptual meaning of the concepts it measures. This limitation could introduce bias in the instrument development process. | No changes have been made to the manuscript |
| 11 | 11. P. 19: The authors mention that primigravida showed a trend towards significant. However, there are many other instances of the p-value being above the significance level, so I am unsure why the authors single out this one. | Thank you for highlighting it. We have clarified the meaning of ‘trend’ towards significance (p value between 0.05 and 0.099) and now included all associated variables with p values which fall in this range in our discussion. | Please see “Discussion” section;  Page 37, line 688-689  Page 38, line 703-706 |
| 12 | 12. Table 4: I would encourage the authors to also report – at minimum – the confidence levels. | Thank you for this comment. As suggested, 95% Confidence Intervals have now been added to the table. | Please see “Table 5” on page 32-34. |
| 13 | 13. Table 4: Why have the authors categorized mother’s age into three categories? Continuous variables retain more power, so is there a specific reason why it is handled as a categorical variable instead? | Thank you for your comment. The three categories: <25 refers to young, 25-34 relatively most fertile age in Pakistan, 35-46 are relatively older women. We wanted to see if there is any association of maternal age with bonding.  As you have highlighted we have also included an analysis of maternal age as a continuous variable. | Please see “Table 5” on page 32 |
| 14 | 14. P. 19 “We used pairwise correlation to assess the correlation between constructs…”: Do the authors mean the subdomains? And I cannot find any report of these analyses in the manuscript. | Thank you – we have expanded on the results of the pairwise correlation tests between domains in the text and now signposted to the results in Table 4. Whilst interchangeable for the purposes of this research, we have nonetheless replaced ‘constructs’ with ‘domains’ here to reduce confusion. | Please see “Results” section, Page 24. Line 536-540  Please see Table 4a, page 30 |
| 15 | Discussion  15. P. 27: The authors mention existing models by Goulet and Corden, but they haven’t been introduced earlier in the background. This is also related to my earlier comment that I am missing a review of the literature on maternal bonding from the authors. | Thank you for this comment. Literature review was based on the existing tools and frameworks used in such tools. We did have mentioned Condon in the review because his framework was used in his tool development. As there was no tool based on Goulet’s framework, we didn’t include it in the review. But as you have highlighted it, we have added details related to Goulet’s framework as well. We have also added their references too;  18. Condon JT, Corkindale CJ. The assessment of parent-to-infant attachment: Development of a self-report questionnaire instrument. Journal of Reproductive and Infant Psychology. 1998;16(1):57-76.  22. Oates J, Gervai J. Mothers' perceptions of their infants. Journal of Prenatal & Perinatal Psychology & Health. 2019;33(4):282-300.  25. Goulet C, Bell L, St-Cyr D, Paul D, Lang A. A concept analysis of parent-infant attachment. J Adv Nurs. 1998;28(5):1071-81. Epub 1998/12/05. doi: 10.1046/j.1365-2648.1998.00815.x. PubMed PMID: 9840879.  In addition to this, we have also added some content related to historical aspects/literature related to bonding and also about attachment.  We have also included relevant references in the manuscript as listed below;  29. Bowlby J. The nature of the child's tie to his mother. Int J Psychoanal. 1958;39(5):350-73. Epub 1958/09/01. PubMed PMID: 13610508.  30. Ainsworth MDS, Blehar MC, Waters E, Wall SN. Patterns of attachment: A psychological study of the strange situation. Hillsdale, NJ: Erlbaum; 1978.  31. Klaus MH, Kennell JH. Mothers separated from their newborn infants. Pediatr Clin North Am. 1970;17(4):1015-37. Epub 1970/11/01. doi: 10.1016/s0031-3955(16)32493-2. PubMed PMID: 4922925.  32. Klaus MH, Jerauld R, Kreger NC, McAlpine W, Steffa M, Kennel JH. Maternal attachment. Importance of the first post-partum days. N Engl J Med. 1972;286(9):460-3. Epub 1972/03/02. doi: 10.1056/nejm197203022860904. PubMed PMID: 5009748.  28. Ettenberger M, Bieleninik Ł, Epstein S, Elefant C. Defining Attachment and Bonding: Overlaps, Differences and Implications for Music Therapy Clinical Practice and Research in the Neonatal Intensive Care Unit (NICU). Int J Environ Res Public Health. 2021;18(4). Epub 2021/02/14. doi: 10.3390/ijerph18041733. PubMed PMID: 33579015; PubMed Central PMCID: PMCPMC7916808. | Please see introduction, page 5, line: 106-117  Please see “References” reference no. 18, 22, 25  Please see introduction, page 6-7, line: 139-150 |
| 16 | 16. As a limitation, I think the authors should address the fact that both the exploratory and confirmatory factor analyses were done in the same sample. | Thank you. We have added this as a limitation to the Discussion section | Please see “Discussion” section, page 39, line: 742-744 |
| 17 | 17. P. 29 “It may also be helpful to further consider the value of the first and second order model options”: Interesting, could the authors expand on this point? | We apologise for this – the sentence was based on an earlier analysis showing more similarity between the models performed prior to standard item reduction procedures, we have now removed this. | Please see “Discussion” section, page 37, line:682-683 |
| 18 | 18. P. 29-30: Could the authors give some interpretations on why they don’t find any significant associations with the cognitive subdomain? | Thanks for your comments. The findings in Table 5 indicate that the cognitive domain is unaffected by both child and maternal characteristics. This domain focuses on maternal perception and higher mental processes such as problem solving and maternal sensitivity and responsiveness to child’s needs. Existing literature indicates that maternal sensitivity and responsiveness may not always be impacted by their mental health^51,52^, which could explain the lack of significant association observed in our study.  51. Huang ZJ, Lewin A, Mitchell SJ, Zhang J. Variations in the relationship between maternal depression, maternal sensitivity, and child attachment by race/ethnicity and nativity: findings from a nationally representative cohort study. Matern Child Health J. 2012;16(1):40-50. Epub 2010/11/26. doi: 10.1007/s10995-010-0716-2. PubMed PMID: 21107669.  52. Amankwaa LC, Pickler RH, Boonmee J. Maternal responsiveness in mothers of preterm infants. Newborn and Infant Nursing Reviews. 2007;7(1):25-30.  We have also added this interpretation and the references in the manuscript. | Please see ‘Result’ section, page 25, line: 548-553  Please see ‘References’ reference no. 51 and 52 |
| 19 | 19. P. 30: I find it unclear whether the authors think that their instrument is valid to assess bonding up to one year postpartum. As the authors have only tested the instrument at one time point, I would be cautious not to overstate the use of the instrument. | Thank you so much for this comment. We developed the instrument to be applicable for one year postpartum. We have added the clarification in the manuscript that current study collected data from women at 42 days postpartum and have recommended that it be tested at other time points post delivery. | Please see ‘Discussion’ page 38, line: 712-718 |
| 20 | Other comments  20. The manuscript would benefit from additional proof-reading. There are several overly long sentences and verb tenses in the same sentence that makes reading challenging. | Thank you for highlighting it. We have proofread the manuscript. | Please see the manuscript |
