# Supplementary material for: Postpartum maternal bonding scale: Development and validation in a low- and middle- income country setting
Source: PLoS One. 2025 Apr 21;20(4):e0317936. doi: 10.1371/journal.pone.0317936 (PMC12011246; doi:10.1371/journal.pone.0317936)
Supplement: Attachment — Submitted filename: Response to reviewers comments_Dec.docx [file pone.0317936.s007.docx]

**Point by point response to reviewers’ comments**

We are thankful to the reviewers for their comments. Following are point by point response to the comments of reviewer 1 and 2.

| **S.No.** | **Reviewer’s Comment** | **Authors’ Response** | **Suggested changes incorporated**  **(Page and line no.). Please see revised manuscript with track changes** |
| --- | --- | --- | --- |
| **Reviewer 1** | | | |
|  | Accepted. No need more revision. All comments have been addressed. Manuscript can be published in PLOS ONE. | Thank you so much for approving our manuscript for publication. We are grateful for your encouraging and insightful comments |  |
| **Reviewer 2** | | | |
|  | Thank you to the authors for their thorough responses to my comments. I believe that the paper has been strengthened considerably. My one remaining note is that although the authors have proof-read the manuscript, I would recommend them to give it one more glance, as there are still some grammatically issues and overly long sentences. For example: | Thank you so much for your kind comments. We have thoroughly proof-read the entire manuscript and all the necessary changes have been made. |  |
| 1 | Introduction, P. 5, l. 104: “Of the nine studies identified” instead of “Of nine studies identified” | Thank you for highlighting it. We have made the corrections. | Please see the introduction section, page 5, line 104 |
| 2 | Introduction, P. 7, l. 155: “It generally may occur” instead of “It generally may occurs” | Thank you. We have corrected it. | Please see the introduction section, page 7, line 154 |
| 3 | Results, P. 23: Ll. 505-513 is all one sentence and too long to be easily read and understood. | Many thanks. We have made the corrections | Please see the ‘Results’ section, page 23, lines 507-514 |
| 4 | Results, P. 24, l. 526: It should be “Correlations” not “Correlation” | Thanks. We have corrected it. | Please see ‘Results’ section, page 23, line 508 |
| 5 | Results, P. 24, l. 535: “linear regression models” instead of “regression methods” | Thank you. We have made the corrections | Please see ‘Results’ section, page 23, line 518 |
| 6 | Discussion, P. 38, L. 712: “…phenomenon of bonding to be matured” instead of “…phenomenon of bonding to matured” | Many thanks. We have corrected it. | Please see ‘Discussion’ section, page 38, line 678 |
| 7 | Discussion, P. 39, Ll. 737: “…attaining a score of zero…” instead of “…attaining zero score…” | Thanks. We have made the corrections. | Please see ‘Discussion’ section, page 39, line 702 |
| 8 | Discussion, P. 40, Ll. 750: Again, I think that the authors should be more cautious in their language. The study may be replicated in other settings, but it is not certain that it can – we would need to conduct these replication studies before we are sure that the study can be replicated. | Thank you for your comment. As suggested, we have made the correction. | Please see ‘Discussion’, page 40, line 712 |
